# Dataset comparing the growth of fodder crops and soil structure dynamics in an industrial biosludge amended arid soil

**DOI:** 10.1016/j.dib.2020.106088

**Published:** 2020-07-25

**Authors:** Reginald B. Kogbara, Wubulikasimu Yiming, Srinath R. Iyengar, Udeogu C. Onwusogh, Karim Youssef, Marwa Al-Ansary, Parilakathoottu A. Sunifar, Dhruv Arora, Ali Al-Sharshani, Osman A.E. Abdalla, Hayel M. Al-Wawi

**Affiliations:** a Mechanical Engineering Program, Texas A&M University at Qatar, P.O. Box 23874, Education City, Doha, Qatar; b Qatar Shell Research & Technology Center, QSTP LLC, Doha, Qatar; c Department of Agricultural Research, Ministry of Municipality & Environment, Doha, Qatar; d Shell Australia Pty Ltd., 275 George Street, Brisbane, QLD 4000, Australia

**Keywords:** Alfalfa, Buffel grass, Days to flowering, Fresh weight biomass, Gas-to-liquid biosludge, Plant height, Porosity, Soil conditioner

## Abstract

The dataset in this work compares the response of two fodder crops, alfalfa (*Medicago sativa*) and buffel grass (*Cenchrus ciliaris*), to industrial biosludge amendment of an arid soil in the State of Qatar. It also evaluates the response of soil structure parameters in the biosludge-amended soils containing the different fodder crops. The dataset relates to our previously published works detailed subsequently. The underlying data comparing the water storage capacity and pore structure evolution of the planted soils treated with 0.75, 1.5, and 3% biosludge contents, which showed good outcomes in the companion articles, alongside soil only and soil-fertilizer controls, are presented. These are shown in terms of the percentage of irrigation water leached, and variations in the logarithmic mean T_2_ (i.e., T2LM - a proxy for mean pore size) and cumulative porosity, respectively. Data on plant growth parameters such as the number of days to flowering, plant height, and aboveground fresh biomass weight in individual replicates of the different treatments as a percentage of the soil-fertilizer control are also shown. The dataset shows the different responses of both plants and the planted soils to amendments with industrial biosludge from the wastewater treatment plant of a gas-to-liquid (GTL) plant.

**Specifications table**

**Table:** 

**Subject**	*Environmental Science*
**Specific subject area**	*Waste Management and Disposal*
**Type of data**	*Figures, Charts and Tables*
**How data were acquired**	*Pot experiments, field measurements, Magritek 2* *MHz nuclear magnetic resonance (NMR) rock core analyzer.*
**Data format**	*Raw, analyzed and calculated*
**Parameters for data collection**	*Direct field measurements and determination of leachate volume; plant performance parameters such as the number of days to flowering, plant height, and aboveground fresh biomass weight; NMR measurements of T_2_ distribution, logarithmic mean T_2_ (*i.e.*, T2LM) and cumulative porosity. The field data were collected from three replicate pots in a given treatment. The experimental site for field data collection has a latitude of 25.82191°N and a longitude of 51.33107°E. The data were collected at the initial (before planting) and final growth stages (12 months after planting).*
**Description of data collection**	*Leachate formed from irrigation water were collected in clean glass bottles through the collection valve of the pots used and the total volume collected in a given period recorded. The NMR instrument mentioned above was used to determine the T_2_ distribution, logarithmic mean T_2_ (*i.e.*, T2LM), and cumulative porosity. Growth parameters of alfalfa and buffelgrass, such as the number of days to flowering and plant height, were determined by field observation of the plants and direct measurement, respectively. The aboveground fresh biomass weight was collected at about 5* *cm above ground level during each cut.*
**Data source location**	*Ministry of Municipality and Environment Research Farm, Rawdat Al-Faras, Al Khor Municipality, Qatar.*
**Data accessibility**	*Data is within this article, and the raw data is deposited on Mendeley at:* https://data.mendeley.com/datasets/n72nn77nvx/draft?*a* = 4188aa3b-4b85–4bbe-8b5c-706c604c5fc7
**Related research article**	R.B. Kogbara, W. Yiming, S.R. Iyengar, U.C. Onwusogh, K. Youssef, M. Al-Ansary, P.A. Sunifar, D. Arora, A. Al-Sharshani, O.A.E. Abdalla, Recycling industrial biosludge for buffel grass production in Qatar: Impact on soil, leachate and plant characteristics, *Chemosphere*, 247 (2020) 125,886, https://doi.org/125,810.121016/j.chemosphere.122020.125886 [1].

**Value of the data**

• The dataset provides information on the different responses of two fodder crops, namely alfalfa and buffel grass, and the planted arid soils amended with industrial biosludge from the wastewater treatment plant of a Gas-to-Liquids (GTL) plant.
• The dataset will help researchers, agricultural scientists, civil/environmental engineers, and environmental management practitioners. It can help improve their understanding of the effects of the GTL biosludge amendment on soil structural properties and how it varies with different plants grown in arid soils.
• The dataset can serve as a reference point for planning field trials evaluating the growth performance of diverse (forage or industrial) crops in biosludge-amended arid soils.
• The dataset is useful as it provides valuable scientific information that can be leveraged to improve agricultural productivity in a region with challenging soil and climatic conditions.
• The dataset generated from the NMR measurements performed in this work can help young researchers understand how to employ the NMR equipment to evaluate soil pore structure parameters.

## 1 Data description

**Fig 1:**
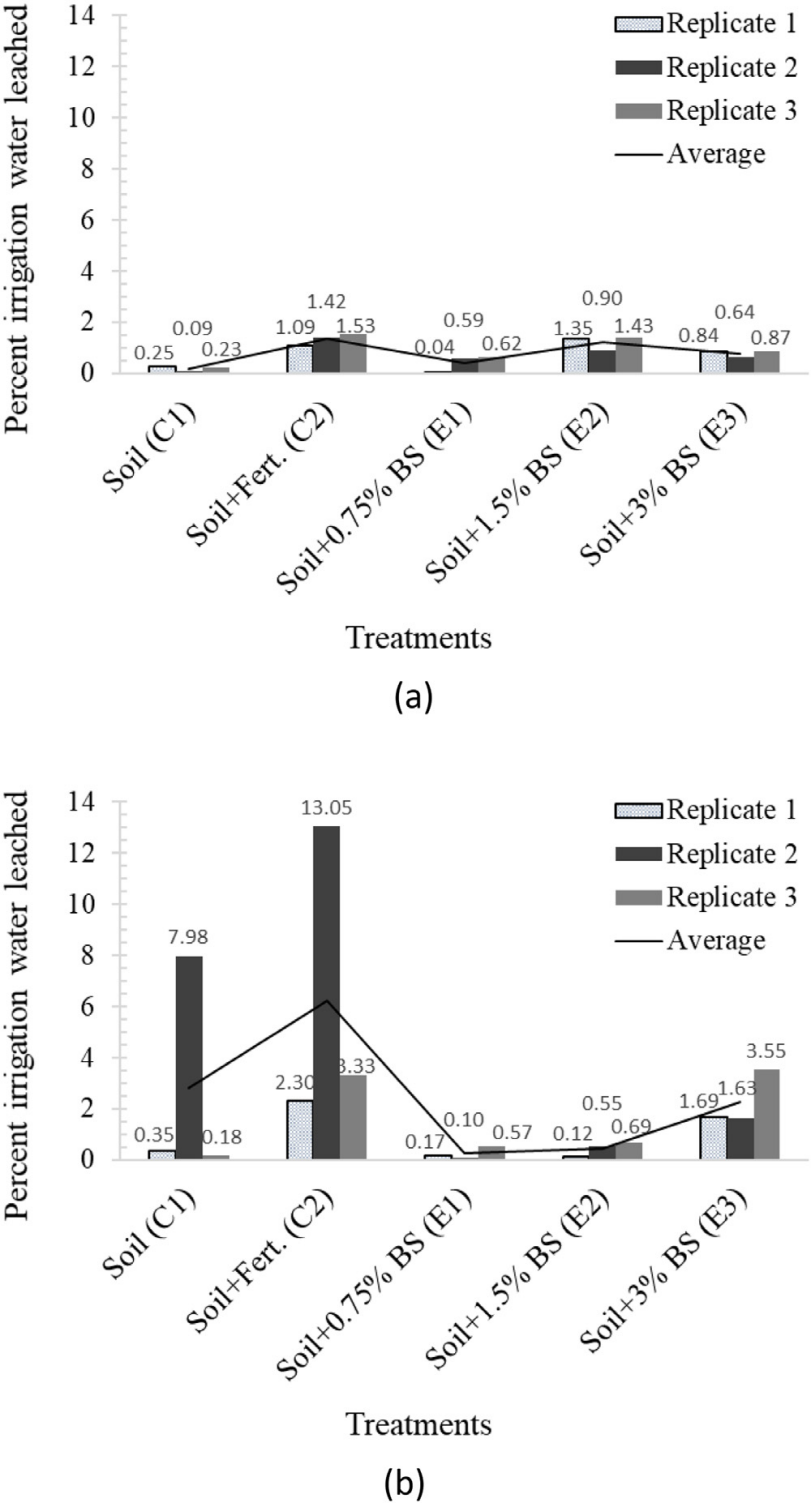
The percentage of irrigation water leached from the soils during the 1-year study period in treatments with (a) alfalfa, and (b) buffel grass. *Note: Alfalfa treatments received 246* *L of water, while buffel grass treatments received 123* *L during the 1-year period*.

**Fig. 2:**
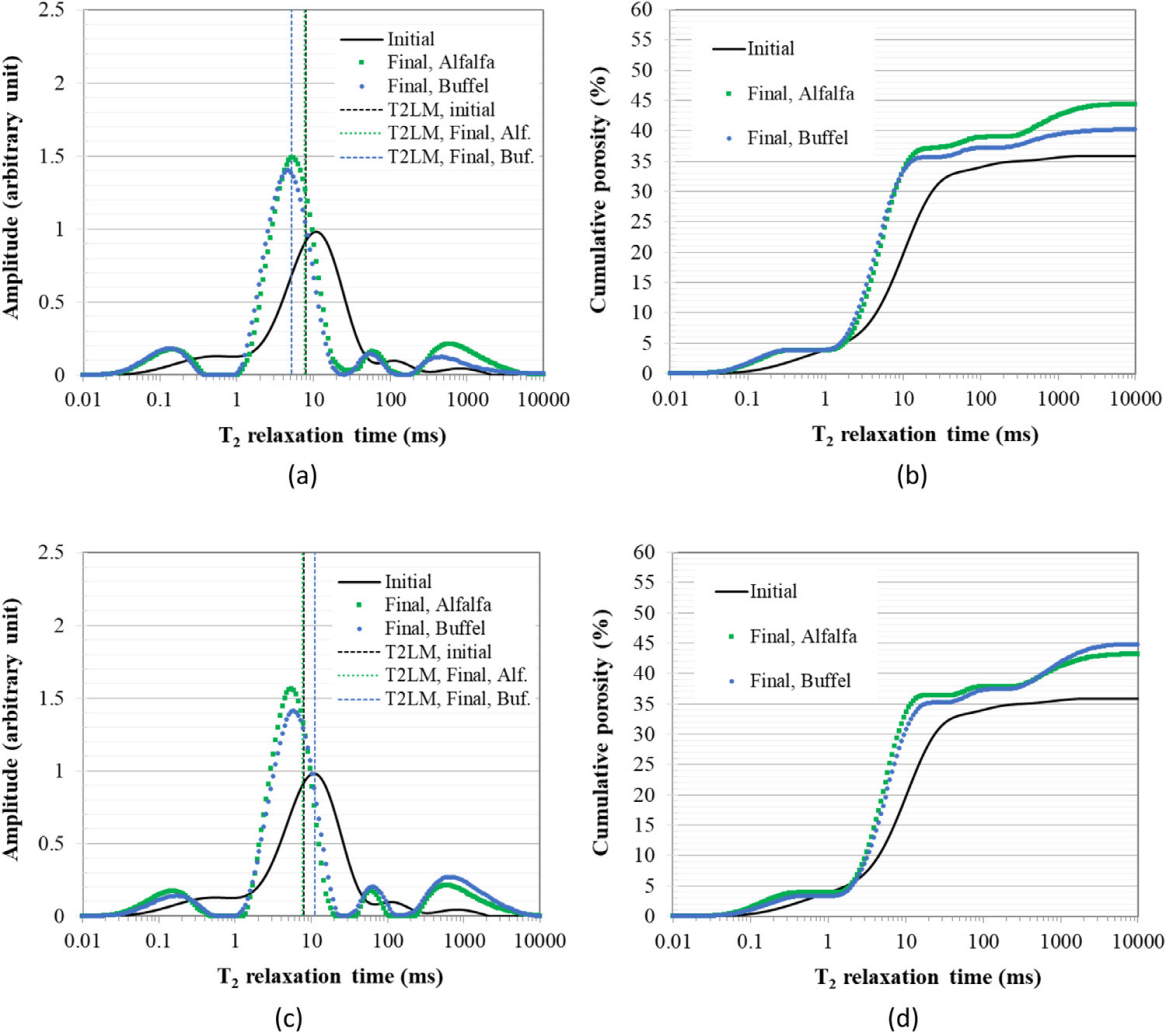
NMR-determined T_2_ distribution and cumulative porosity for representative samples of the controls with (a) and (b) Soil only – C1, and (c) and (d) Soil-fertilizer - C2, respectively, at the initial (before planting) and final growth stages. *Note: T2LM – logarithmic mean T_2_, Alf. – Alfalfa, Buf. Buffel grass. The T2LM is indicated as vertical dotted lines in the T_2_ distribution spectra, similar to how the Prospa software outputs it, although it is done for several specimens here.*

**Fig. 3:**
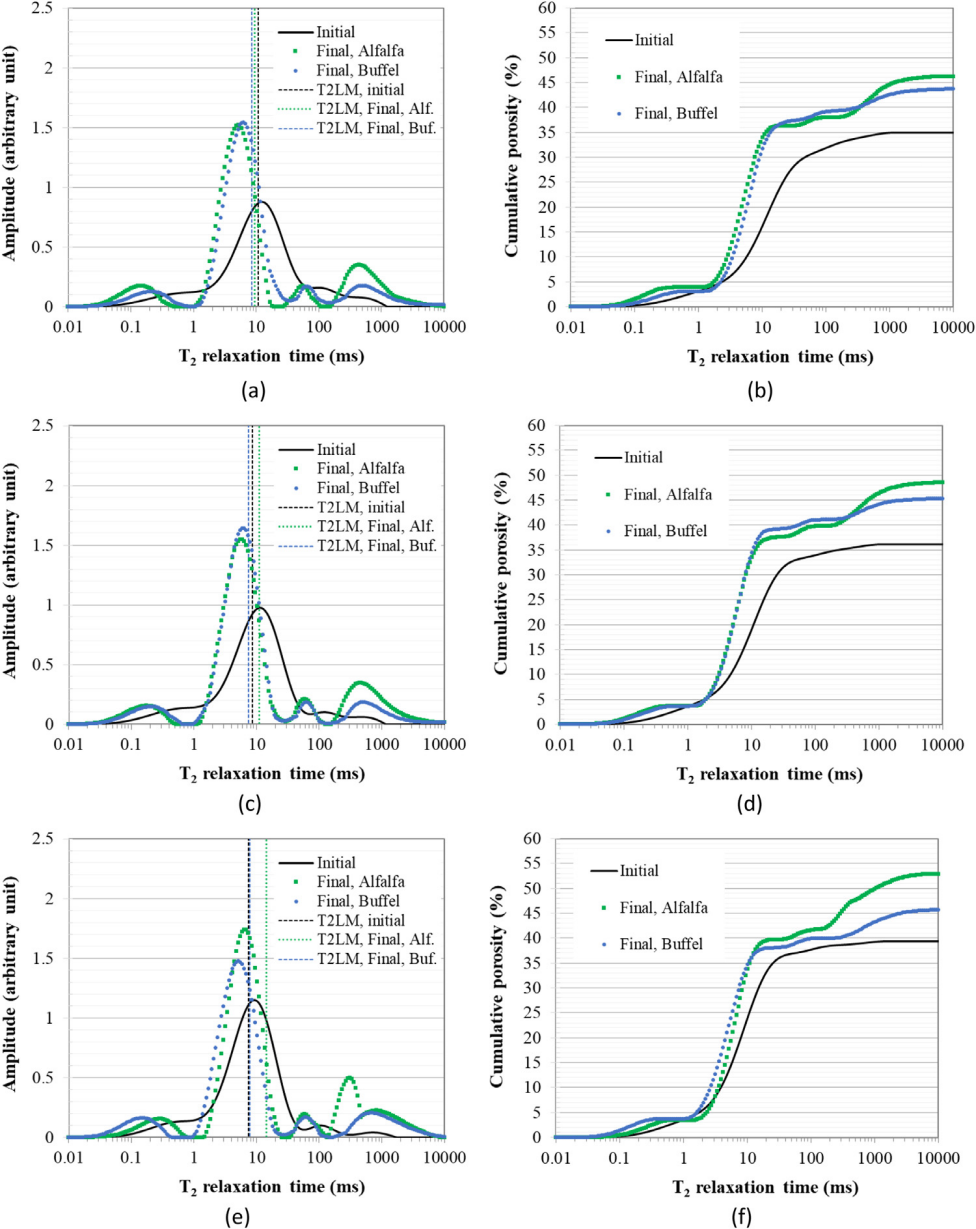
NMR-determined T_2_ distribution and cumulative porosity for representative samples of the treatments with (a) and (b) Soil + 0.75% BS – E1, (c) and (d) Soil + 1.5% BS – E2, and (e) and (f) Soil + 3% BS – E3, respectively, at the initial (before planting) and final growth stages. *Note: T2LM – logarithmic mean T_2_, Alf. – Alfalfa, Buf. Buffel grass, BS - biosludge. The T2LM is indicated as vertical dotted lines in the T_2_ distribution spectra, similar to how the Prospa software outputs it, although it is done for several specimens here.*

The percentage of irrigation water leached is shown in Fig. 1 for alfalfa and buffel grass as a proxy for the water storage capacity of the soils [2]. This is shown for each of the three replicates of planted soils in the five treatments considered in this work, namely soil plus 0.75, 1.5, and 3% biosludge contents, which showed good outcomes in our previously published works [1,3], alongside soil only and soil-fertilizer controls. In contrast, related data was presented as average leachate volume, and average volume flow rate with mean and standard deviation values in the companion articles [1,3]. Fig. 2, Fig. 3 demonstrate the change in the logarithmic mean T_2_ (i.e., T2LM - a proxy for mean pore size) and cumulative porosity between the initial (before planting) and final growth stages for both plants. The data are replotted from the spectra for T_2_ distribution and cumulative porosity provided by the NMR instrument for representative samples of the soils in the different treatments and used for comparisons for both plants. The data for the above soil structure parameters serves to highlight the influence of the biosludge amendment of the arid soil on soil structure dynamics in planted soils with the different crops. Hence, it compares the selected biosludge treatments with the soil-only control, and the soil-fertilizer control - a typically used method for farming in Qatar.

**Fig. 4:**
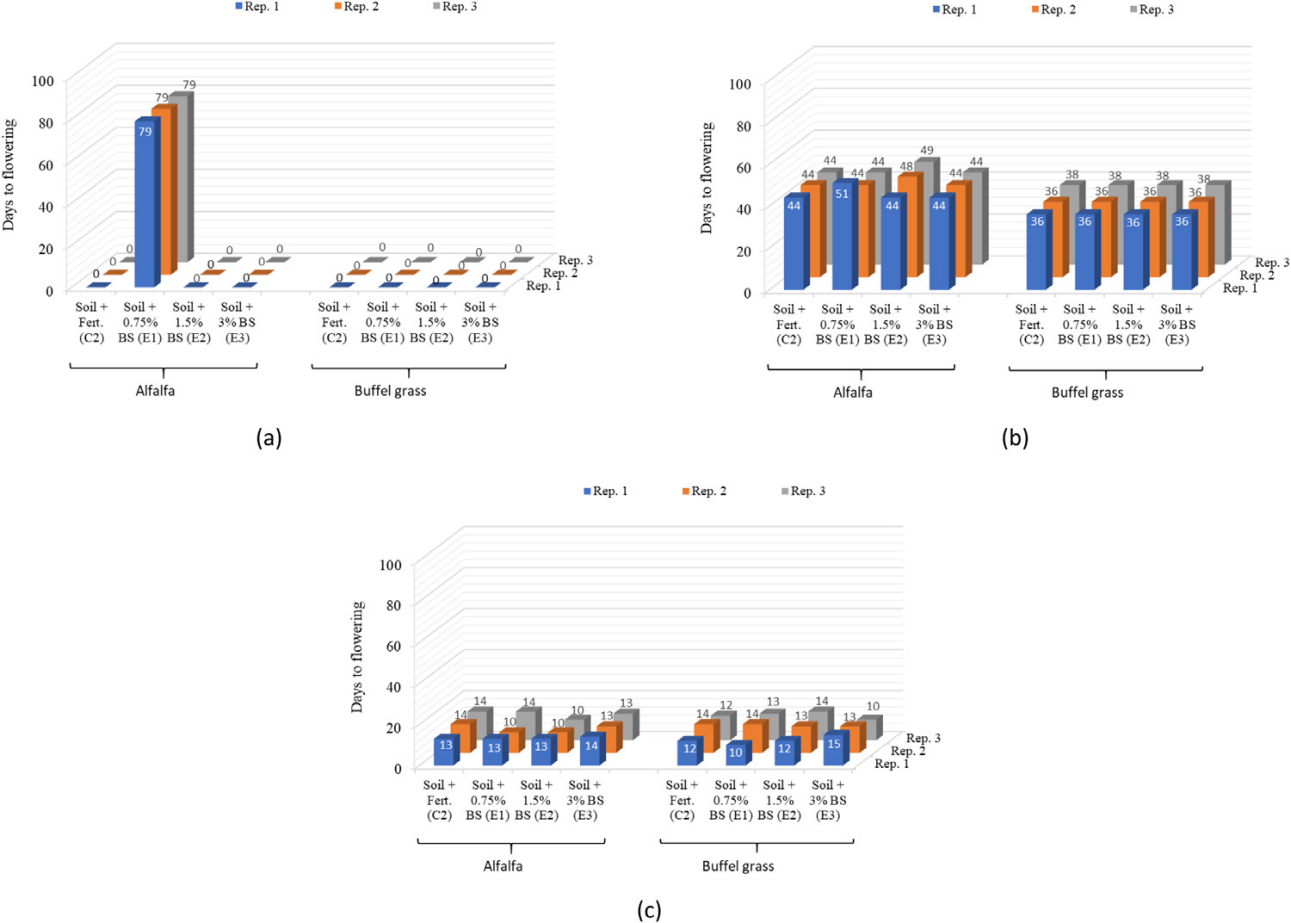
The number of days to flowering in the different treatments with alfalfa and buffel grass at the (a) first cut, (b) second cut, and (c) third cut. *Note: Rep. – Replicate, Fert. – fertilizer, BS: biosludge. There was no flowering in the control/treatments with 0 days to flowering at the first cut.*

**Fig. 5:**
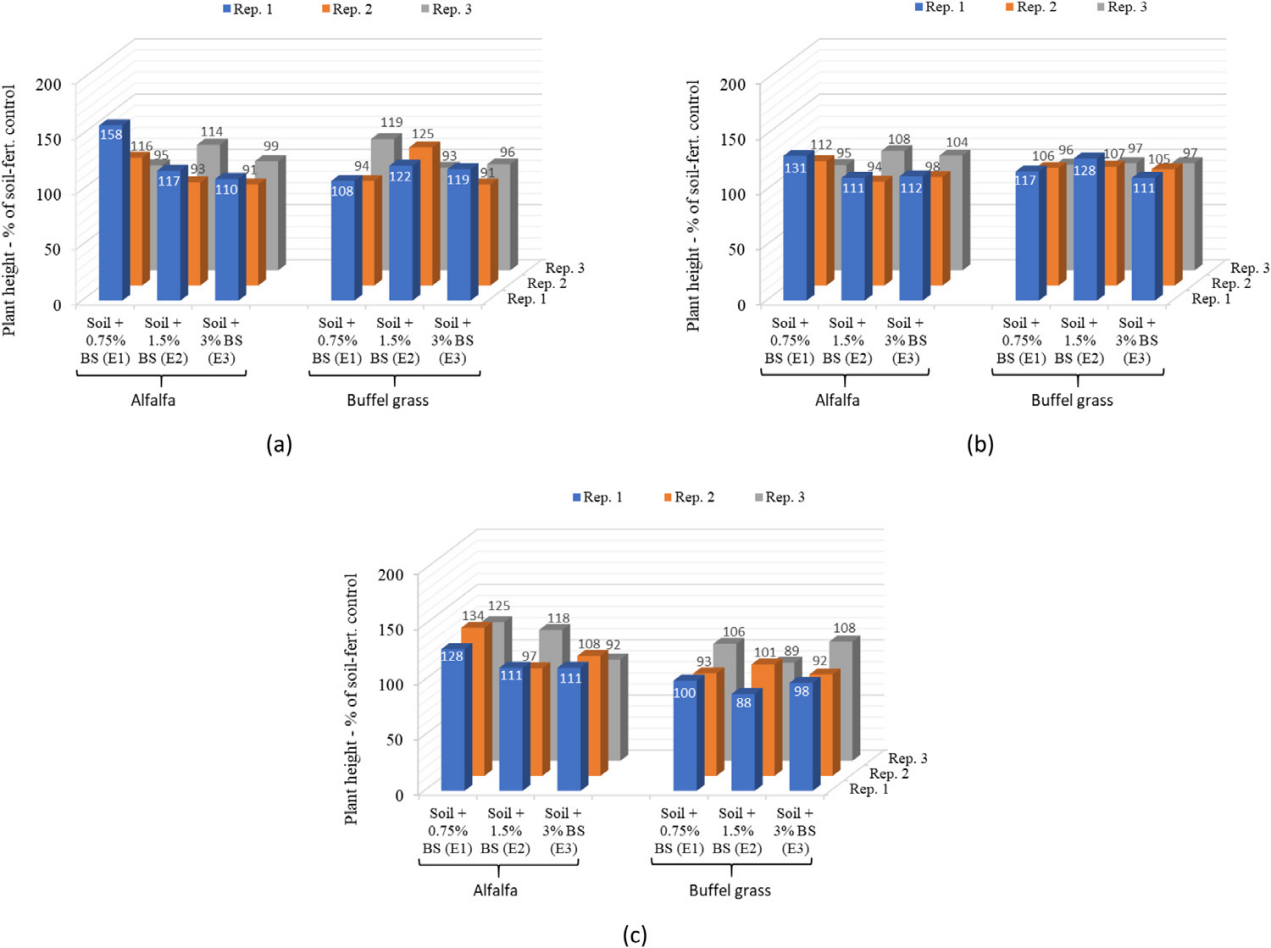
Plant height as a percentage of the soil-fertilizer control in the different biosludge treatments with alfalfa and buffel grass at the (a) first cut, (b) second cut, and (c) third cut. *Note: Rep. – Replicate, Fert. – fertilizer, BS: biosludge*.

**Fig. 6:**
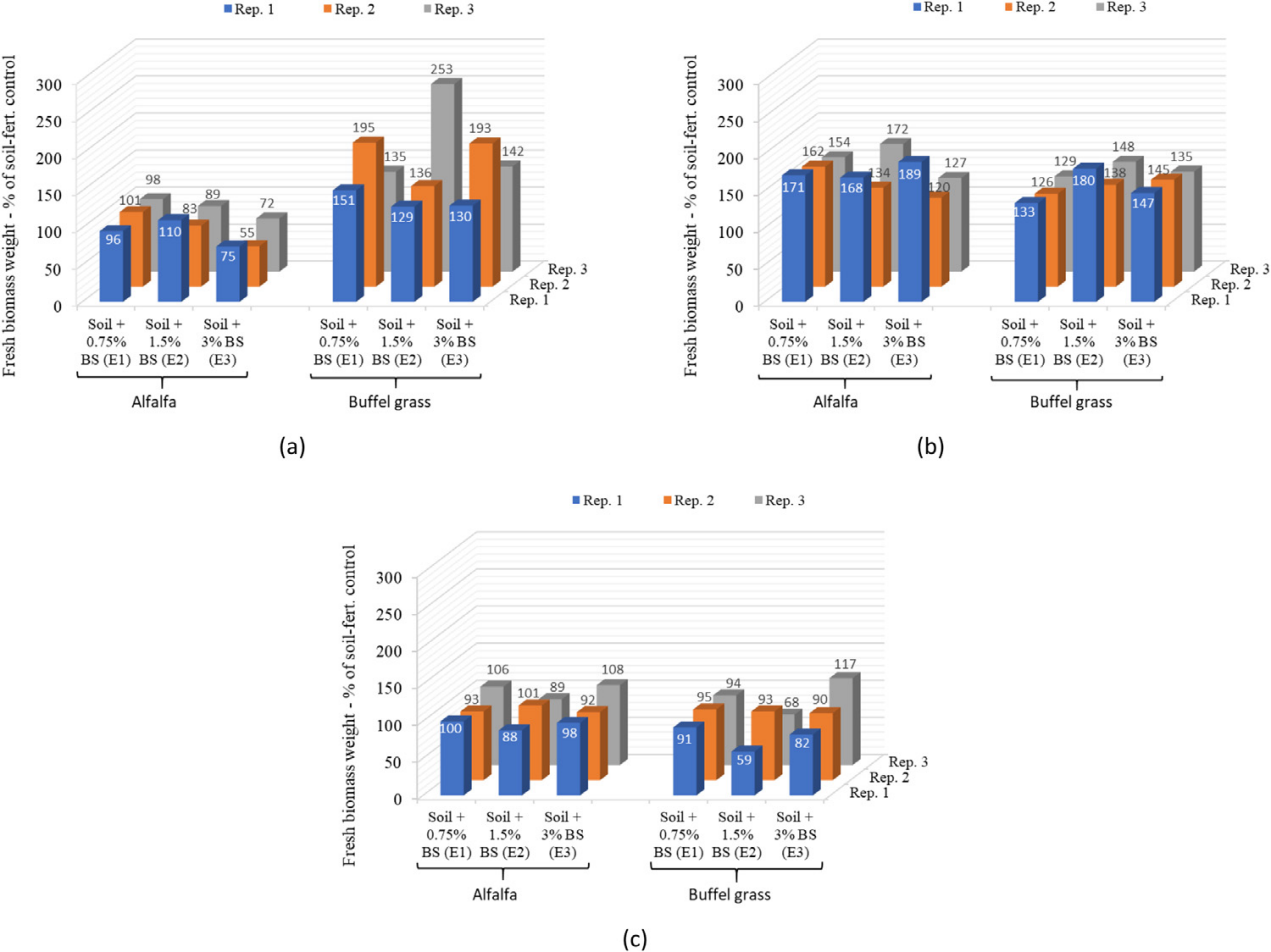
Fresh biomass weight as a percentage of the soil-fertilizer control in the different biosludge treatments with alfalfa and buffel grass at the (a) first cut, (b) second cut, and (c) third cut. *Note: Rep. – Replicate, Fert. – fertilizer, BS: biosludge*.

Data on plant growth parameters such as the number of days to flowering, plant height, and aboveground fresh biomass weight in individual replicates of the selected biosludge treatments as percentages of the average value in the soil-fertilizer control are shown in Fig. 4, Fig. 5, Fig. 6, respectively. The data for the growth parameters in the soil-only control is not relevant here; hence, not shown. The data are presented in 3D column charts for each of the three plant cuts carried out during the study and directly compare the growth parameters of alfalfa and buffel grass. This differs from the data presented in the companion articles [1,3]. It compares the growth parameters for both crops in each replicate of the selected biosludge treatments considered here, as a percentage of the soil-fertilizer control, which is typically used for farming [4]. In contrast, the absolute values of a given growth parameter in the treatments were presented as means and standard deviations for either crop in the companion articles. Nevertheless, the actual number of days to flowering is used in the comparison for both plants rather than as a percentage of the soil-fertilizer control. The parameter was not reported in the companion articles but was presented differently for alfalfa only in a related data article [5]. The growth parameters dataset shows the different responses of the growth of both fodder crops to the industrial biosludge amendment of the arid soil.

## 2 Experimental design, materials, and methods

The materials and experimental methodology employed in this work have been detailed in the companion articles [1,3]. Hence, only pertinent information is presented in the description of how the dataset in this article was acquired.

### 2.1 Experimental materials

The pot experiments involved three replicate 92 cm long cylindrical pots with 52 cm diameter for each treatment. Each pot had a valve connected at the bottom to permit leachate collection, alongside some gravel (> 2 mm) and fine sand in the bottom layer to prevent clogging and facilitate water movement. The bottom of the pots had a 6,7 degrees slope made by filling them with glass-reinforced plastic at a slight tilt, which enabled the leachate's direction to the water collection valve [6].

Five treatments are considered in this paper, two controls, and three selected biosludge treatments that showed excellent performance in the companion articles. The first is the soil only control (C1). It entailed using a typical soil available in Qatari farms that were obtained from the experimental research farm of the Agricultural Department of Qatar Ministry of Municipality and Environment at Rawdat Al-Faras, Al Khor Municipality. The second treatment is the soil-fertilizer control (C2) in which a commercially available 20–20–20 NPK fertilizer was used with urea. The NPK fertilizer was applied at 100 kg/ha, corresponding to 2.12 g per pot, while the urea was applied at 75 kg/ha, corresponding to 1.59 g per pot. The fertilizer was applied in three doses at 2, 12, and 24 weeks after planting [7,8]. The selected biosludge treatments (E1 – E3) had mixtures of soil and 0.75, 1.5, and 3% biosludge contents. The GTL biosludge used was obtained from a GTL plant in Qatar. The replicate pots in the treatments were arranged in a completely randomized design. A set of the treatments contained alfalfa seedlings, while another set contained Buffel grass seedlings.

### 2.2 Seeding, irrigation and sampling

Alfalfa and Buffel grass seeds were sowed at 1 cm depth at 10 locations in each pot after the pots were irrigated to set the soil columns. The pots were manually irrigated based on the different irrigation requirements of both crops for different months. The pots were irrigated every three days during the winter months and every day in the summer months. In the event of rainfall, the rainfall amount was deducted from the irrigation water for that day. The annual average irrigation water requirement for alfalfa is 2.71 mm/day, with the lowest value (1.3 mm/day) in January and the highest value (5.6 mm/day) in July. Buffel grass has half the irrigation water requirements of alfalfa. This followed the usual irrigation practice of the Qatar Ministry of Municipality and Environment. Soil samples were collected from the pots for initial analysis before seed sowing and at the final-growth stage (12 months) using a tube sampler (auger). Plant samples were collected at each of the three cuts (harvest) conducted during the 1-year study period. All pots were regularly checked for leachate formation. When formed, leachate was collected in clean glass bottles through the collection valve of the pots.

### 2.3 Analysis of plant, soil and leachate parameters

The following describes the methods employed in the analyses of soil, plant and leachate samples from the different treatments. Mixtures of soil and fertilizer/biosludge are simply described as soil in this section for convenience.

*Soil pore structure parameters:* Pore structure parameters, namely, cumulative porosity and T_2_ distribution, were determined using a 2 MHz nuclear magnetic resonance (NMR) rock core analyzer with a 54 mm probe (*Magritek*, New Zealand). The details of the testing method can be found elsewhere [9]. The T_2_ distribution is usually used as a proxy for pore size distribution rather than converting it to the actual pore size distribution since relaxation times are impacted by the presence of paramagnetic species such as Fe. The soil mixtures used here contained significant amounts of Fe originating from the biosludge, as shown in the companion articles [1,3]. The T_2_ relaxation data were determined on a water-saturated soil sample placed in a 20 ml cylindrical plastic container using the Carr-Purcell-Meiboom-Gill (CPMG) sequence. Key parameters employed was a 100 μs echo time, an inter-experimental delay time of 6500 ms and 200 scans. Prospa software (*Magritek*, New Zealand) was then employed to analyze the CPMG decay using the Lawson and Hanson non-negative least square fit method, and output the logarithmic mean T_2_ (T2LM) - a proxy for the mean pore size. The calculation of the T2LM is based on Eq. (1)

(1) $$T2LM=e^{\left(\frac{\sum_{i=1}^{n}a_{i}\ln T_{2i}}{\sum_{i=1}^{n}a_{i}}\right)}$$

Where, *T*2*LM* is the logarithmic mean *T*_2_, while *T*_2*i*_ and *a_i_* are the relaxation times and amplitudes, respectively, in the T_2_ distribution curves.

*Analysis of percentage irrigation water leached:* The entire leachate volume drainable via the collection valve of the pots was collected during each sampling when leachates formed, and the total amount obtained in a given period recorded. The total leachate volume recorded throughout the 1-year study period was then expressed as a percentage of the total volume of irrigation applied. Although the leachate collection period lasted much longer than the 1-year study period, as shown in one of the companion articles [3], only the leachate collected during the study period is considered to allow a consistent comparison with the irrigation applied.

*Number of days to flowering*: The days to flowering was recorded as the number of days from the planting date to the opening of the first flower for each crop [10].

*Plant height*: This was determined by measuring the distance from the soil level to the terminal bud of the longest stem on that plant [11].

*Aboveground fresh biomass weight*: The fresh biomass weight was taken from samples of 10 plants. The samples were obtained using a stainless-steel grass shear to snip plants at about 5 cm above ground level during each of the three cuts carried out [12]. The cuts were carried out on the plants at 3, 6 and 7 months after planting in line with the normal agronomic practice in Qatar.

## Conflict of interest

The authors declare that they have no known competing financial interests or personal relationships that could have appeared to influence the work reported in this paper.

## Credit author statement

**R.B. Kogbara**: Conceptualization, Methodology, Investigation, Formal analysis, Writing – original draft. **W. Yiming**: Conceptualization, Investigation, Data curation, Visualization. **S.R. Iyengar**: Conceptualization, Methodology, Data curation, Writing – review & editing. **U.C. Onwusogh**: Conceptualization, Methodology, Data curation, Writing – review & editing, Project administration. **K. Youssef**: Validation, Investigation, Visualization. **M. Al-Ansary**: Conceptualization, Methodology, Supervision. **P.A. Sunifar**: Validation, Investigation. **D. Arora**: Methodology, Writing – review & editing, Supervision. **A. Al-Sharshani**: Writing – review & editing, Project administration**. O.A.E. Abdalla**: Conceptualization, Methodology, Validation, Investigation, Data curation. **H.M. Al-Wawi**: Supervision, Project administration.

## Declaration of Competing Interest

The authors declare that they have no known competing financial interests or personal relationships that could have appeared to influence the work reported in this paper.

## References

[1] Kogbara R.B., Yiming W., Iyengar S.R., Onwusogh U.C., Youssef K., Al-Ansary M., Sunifar P.A., Arora D., Al-Sharshani A., Abdalla O.A.E. (2020). Recycling industrial biosludge for buffel grass production in Qatar: Impact on soil, leachate and plant characteristics. Chemosphere.

[2] Pantini S., Verginelli I., Lombardi F. (2014). A new screening model for leachate production assessment at landfill sites. Int. J. Environ. Sci. Technol.

[3] Kogbara R.B., Yiming W., Iyengar S.R., Abdalla O.A.E., Al-Wawi H.M., Onwusogh U.C., Youssef K., Al-Ansary M., Sunifar P.A., Arora D. (2020). Effect of gas-to-liquid biosludge on soil properties and alfalfa yields in an arid soil. J. Clean. Prod..

[4] Stocker R.K., Haller W.T. (1999). Residual effects of herbicide-treated Eichhornia crassipes used as a soil amendment. Hydrobiologia.

[5] Kogbara R.B., Yiming W., Iyengar S.R., Onwusogh U.C., Youssef K., Al-Ansary M., Sunifar P.A., Arora D., Abdalla O.A.E., Al-Wawi H.M. (2020). Dataset on the influence of gas-to-liquid biosludge on arid soil properties and growth performance of alfalfa. Data Brief.

[6] Khire M.V., Mukherjee M. (2007). Leachate injection using vertical wells in bioreactor landfills. Waste Manage..

[7] Ayotamuno J.M., Kogbara R.B. (2007). Determining the tolerance level of Zea mays (maize) to a crude oil polluted agricultural soil. Afr. J. Biotechnol.

[8] Ayotamuno J.M., Kogbara R.B., Agoro O.S. (2009). Biostimulation supplemented with phytoremediation in the reclamation of a petroleum contaminated soil. World J. Microbiol. Biotechnol.

[9] Kogbara R.B., Iyengar S.R., Grasley Z., Masad E.A., Zollinger D.G. (2015). Non-destructive evaluation of concrete mixtures for direct LNG containment. Mater. Des..

[10] González A.M., Yuste-Lisbona F.J., Saburido S., Bretones S., De Ron A.M., Lozano R., Santalla M. (2016). Major contribution of flowering time and vegetative growth to plant production in common bean as deduced from a comparative genetic mapping. Front. Plant Sci..

[11] Barney G., Massengale M.A., Dobrenz A.K. (1974). Effect of seeding rate and harvest management on yield and stand persistence in Alfalfa. J. Ariz. Acad. Sci..

[12] Hedlund K., Santa Regina I., Van der Putten W.H., Lepš J., Díaz T., Korthals G.W., Lavorel S., Brown V.K., Gormsen D., Mortimer S.R., Rodríguez Barrueco C., Roy J., Smilauer P., Smilauerová M., Van Dijk C. (2003). Plant species diversity, plant biomass and responses of the soil community on abandoned land across Europe: idiosyncracy or above-belowground time lags. Oikos.

